# Methylhydrazine Lone‐Pair Engineering for Polar Lead‐Free Perovskite Enables Self‐Powered X‐Ray Detection

**DOI:** 10.1002/smsc.202400508

**Published:** 2025-03-11

**Authors:** Ruiqing Li, Jianbo Wu, Zeng‐Kui Zhu, Yaru Geng, Xinling Li, Yifei Wang, Bohui Xu, Zheshuai Lin, Junhua Luo

**Affiliations:** ^1^ State Key Laboratory of Structural Chemistry Fujian Institute of Research on the Structure of Matter Chinese Academy of Sciences, Fuzhou Fujian 350002 P. R. China; ^2^ Shandong Provincial Key Laboratory of Chemical Energy Storage and Novel Cell Technology School of Chemistry and Chemical Engineering Liaocheng University Liaocheng Shandong 252000 China; ^3^ School of Chemical Sciences College of Materials Science and Opto‐electronic Technology University of Chinese Academy of Sciences Beijing 100049 China; ^4^ Functional Crystals Lab Technical Institute of Physics and Chemistry Chinese Academy of Sciences Beijing 100190 China

**Keywords:** lead‐free, lone‐pair engineering, low detection limit, polar A_3_Bi_2_I_9_‐type perovskite, self‐powered X‐ray detection

## Abstract

Lead‐free A_3_Bi_2_I_9_‐type perovskites demonstrate excellent performance in direct X‐ray detection owing to their high bulk resistivity and reduced ion migration. However, the reported centrosymmetric A_3_Bi_2_I_9_ can only operate with external voltage, inevitably resulting in energy consumption and bulky monolithic circuits, limiting their further development. Herein, exploiting the methylhydrazine (MHy) cation with 2*s* ^2^ lone‐pair electrons (LPEs), a chiral‐polarity perovskite MHy_3_Bi_2_I_9_ are obtained and explored its self‐powered X‐ray detection properties. Where MHy forms the strong hydrogen bond interaction with the inorganic framework, resulting in the asymmetric Bi_2_I_9_ unit. Meanwhile, the 2*s* ^2^ LPEs contribute to generating MHy dipole moments, leading to spontaneous polarization. On the one hand, spontaneous polarization acts as a driving force to realize the X‐ray‐generated carriers’ separation and transport to acquire self‐powered detection ability. On the other hand, the reduced noise current and dark current under zero bias further increase the signal‐to‐noise ratio and lower the detection limit. Notably, the MHy_3_Bi_2_I_9_ single‐crystal‐based detector displays a considerable sensitivity (106 μC Gy^−1^ cm^−2^) and an ultralow detection limit (55 nGy s^−1^) in self‐powered mode. Herein, new insights for constructing polar lead‐free perovskite and realizing unprecedented A_3_Bi_2_I_9_‐type self‐powered X‐ray detectors are provided.

## Graphical abstract

Utilizing the organic cation MHy with 2*s* ^2^ lone‐pair electrons, the chiral‐polar lead‐free perovskite MHy_3_Bi_2_I_9_ is constructed in the famous A_3_Bi_2_I_9_ system. The symmetry breaking ascribes to the asymmetric transmission from MHy to the Bi_2_I_9_ inorganic skeleton through hydrogen bonding. Exploiting the polarization‐induced bulk photovoltaic effect, the MHy_3_Bi_2_I_9_ detector exhibits self‐powered X‐ray detection with an ultralow detection limit (55 nGy s^−1^).

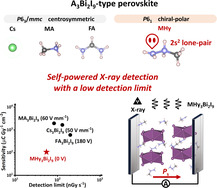

## 1 Introduction

X‐ray detection has attracted increasing attention owing to its wide application in medical diagnostics, environmental monitoring, and security screening.^[^ ^1^, ^2^, ^3^ ^]^ Lead halide hybrid perovskites have recently attracted massive attention in X‐ray detection because of their flexible structural designability and remarkable carrier transport capability.^[^ ^4^ ^]^ For example, MAPbI_3_ (MA = methylamine) single‐crystal(SC) based detector discloses a high sensitivity of about 6218 μC Gy^−1^ cm^−2^.^[^ ^5^ ^]^ Despite this great progress, Pb‐based detectors are confronted with serious pollution and high toxicity. To address this toxicity problem, the development of “green” X‐ray detection is a wise choice.^[^ ^6^ ^]^

Trivalent bismuth (Bi^3+^) cation has a large relative atomic number (*Z* = 83) and resembles Pb^2+^ cation in the electronic configuration, which is a promising alternative for Pb.^[^ ^7^ ^]^ Particularly, owing to the high density, large bulk resistivity, and reduced ion migration, A_3_Bi_2_I_9_‐type perovskite has made great progress in the X‐ray detection region.^[^ ^8^ ^]^ Recently, Zhang et al. reported the X‐ray detection properties of Cs_3_Bi_2_I_9_ SC with high sensitivity (1652 μC Gy^−1^ cm^−2^).^[^ ^9^ ^]^ Further, Liu and co‐workers report the X‐ray detection properties of MA_3_Bi_2_I_9_ SC with a high sensitivity (1947 μC Gy^−1^ cm^−2^).^[^ ^10^ ^]^ Despite this great progress, the reported A_3_Bi_2_I_9_‐type perovskites (Cs_3_Bi_2_I_9_, MA_3_Bi_2_I_9_, FA_3_Bi_2_I_9_, and FA = formamidine) all crystalize in the centrosymmetric space group *P*6_3_/*mmc*, which can only operate under large external electric fields, inevitably results in large energy consumption and high detection limit.^[^ ^11^ ^]^ As for application in medical diagnostics, a low dose rate is desired to avoid X‐ray irradiation of the human body.^[^ ^12^ ^]^ Notably, perovskites with spontaneous polarization can realize the X‐ray‐generated carriers’ separation and transport and support X‐ray detection even at zero bias.^[^ ^13^, ^14^, ^15^, ^16^, ^17^ ^]^ Importantly, under the self‐driven mode, detectors with spontaneous polarization enable suppressed dark current and noise current, thus increasing the signal‐to‐noise ratio (SNR) and lowering the detection limit.^[^ ^18^ ^]^ Recently, exploiting the chirality‐induced polar strategy, (R/S‐PPA)_2_BiI_5_ SC accomplished the X‐ray detection, which disclosed an ordinary sensitivity (31 μC Gy^−1^ cm^−2^) and detection limit (270 nGy s^−1^). (R‐MPA)_4_AgBiI_8_ SC displayed a low detection limit (LoD) (85 nGy s^−1^).^[^ ^19^ ^]^ Unfortunately, the infrequent chiral organic cations hinder the expansion of polar lead‐free perovskite, while the excessive steric hindrance induces a low density, further resulting in unsatisfactory X‐ray performance.

As local asymmetric units, the lone‐pair electrons (LPEs) can induce spontaneous polarization by directional arrangement, thus providing promising in self‐powered detection.^[^ ^20^, ^21^, ^22^ ^]^ Recently, the unique cation MHy exhibits 2*s* ^2^ LPEs in the terminal *N* atom and displays promising performance in constructing polar perovskite.^[^ ^23^, ^24^, ^25^ ^]^ For example, MHyPbBr_3_ crystalized in the polar space group *P*2_1_ owing to the 2*s* ^2^ LPEs while MAPbBr_3_ and FAPbBr_3_ crystalized in the centrosymmetric space group *Pnma* owing to the absence of 2*s* ^2^ LPEs.^[^ ^26^, ^27^, ^28^ ^]^ Benefitting from the polarization, Guan et al. explored the self‐powered X‐ray performance of MHyPbBr_3_ SC, which manifests a LoD of 203 nGy s^−1^.^[^ ^29^ ^]^ However, the corresponding LPEs’ investigation is still blank in the lead‐free perovskite region. Therefore, introducing MHy cation with 2*s* ^2^ LPEs into lead‐free A_3_Bi_2_I_9_‐type perovskites would be a promising way to realize spontaneous polarization and construct high‐sensitive self‐powered X‐ray detectors.

Herein, by exploiting MHy lone‐pair engineering, we successfully obtained a chiral‐polar perovskite with A_3_Bi_2_I_9_‐type characters, MHy_3_Bi_2_I_9_. From the 2*s* ^2^ LPEs, MHy forms a strong hydrogen‐bond interaction with the inorganic framework and results in the Bi_2_I_9_ dipole moments. Meanwhile, the MHy dipole moments form a 6‐fold roto‐inversion along the *c*‐axis, leading to the chirality and polarity, coincident well with the chiral‐polar *P*6_1_ space group. The pyroelectricity measurements verified the polarization characteristics. Notably, integrating the advantages of high density, large bulk resistivity, strong X‐ray absorption, and spontaneous polarization, MHy_3_Bi_2_I_9_ not only displays a high sensitivity up to 5214.7 μC Gy^−1^ cm^−2^ under an electric field but also exhibits a considerable sensitivity of about 106 μC Gy^−1^ cm^−2^ and ultra‐LoD at 55 nGy s^−1^ under self‐powered mode. This work exploits the lone‐pair engineering toward polar lead‐free perovskite and provides a new sight toward “green” self‐powered X‐ray detection.

## 2 Results and Discussion

### 2.1 Crystal Structure

#### 2.1.1 Structure Feature of A_3_Bi_2_I_9_‐type Perovskites

**Figure 1:**
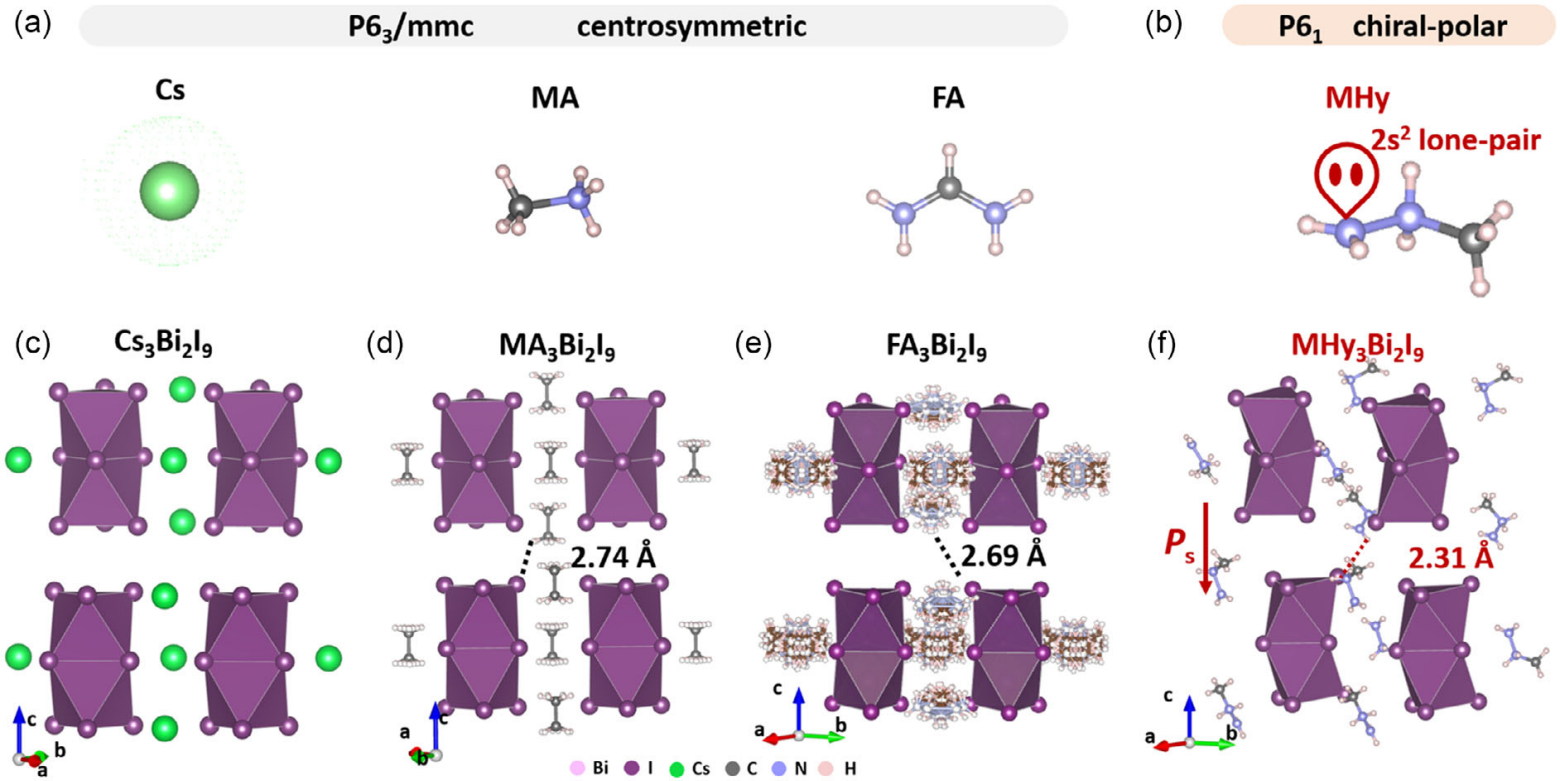
a) The crystal structure of typical A‐cation (Cs, MA, and FA) in A_3_Bi_2_I_9_‐type perovskite. b) The MHy cation with 2*s* ^2^ lone‐pair electrons. The room‐temperature crystal structure of c) Cs_3_Bi_2_I_9_, d) MA_3_Bi_2_I_9_, and e) FA_3_Bi_2_I_9_, which crystalized in the centrosymmetric space group *P*6_3_/*mmc*. f) Exploiting the MHy as A‐cation, MHy_3_Bi_2_I_9_ possesses a strong hydrogen bond interaction and crystalizes in the chiral‐polar space group *P*6_1_ at room temperature. The dotted line refers to the hydrogen bond length between the adjacent layers.

The famous perovskite family, A_3_Bi_2_I_9_, where A‐site cation refers to the cation with small steric hindrance, such as Cs, MA, and FA (**Figure** **1**a). A_3_Bi_2_I_9_‐type perovskite displays an isolated inorganic framework, the BiI_6_ octahedra share a common face to form a Bi_2_I_9_^3−^ binuclear cluster.^[^ ^30^, ^31^ ^]^ Here, we report a new A_3_Bi_2_I_9_‐type perovskite with the novel organic cation MHy. The crystal data have been provided in Table S1–S5, Supporting Information. Notably, the valence electron configuration of the *N* atom is 1*s* ^2^2*s* ^2^2*p* ^3^. In the organic cation MHy^+^, the terminal *N* atom bonds with two *H* atoms and one *N* atom, and remains the stereo‐active 2*s* ^2^ lone‐pair electron, further resulting in the local asymmetric unit (Figure 1b). The lone‐pair activity is verified by the electron localization function (Figure S1, Supporting Information). For comparison, MA^+^ and FA^+^ cations are not equipped with 2*s* ^2^ LPEs. In detail, the *N* atom in MA^+^ cation bonds with three *H* atoms and one *C* atom therefore does not possess unbonded valence electrons. The 2*s* ^2^ lone pair in the unprotonated NH_2_ group of FA cation participates in forming delocalized bonds. Therefore, FA^+^ does not contain stereo‐active 2*s* ^2^ LPEs. A striking contrast is that Cs_3_Bi_2_I_9_, MA_3_Bi_2_I_9_, and FA_3_Bi_2_I_9_ crystalized in the centrosymmetric space group *P*6_3_/*mmc* (Figure 1c–e) while MHy crystalized in the chiral‐polar space group *P*6_1_.

#### 2.1.2 Asymmetric Characteristic in MHy_3_Bi_2_I_9_

**Figure 2:**
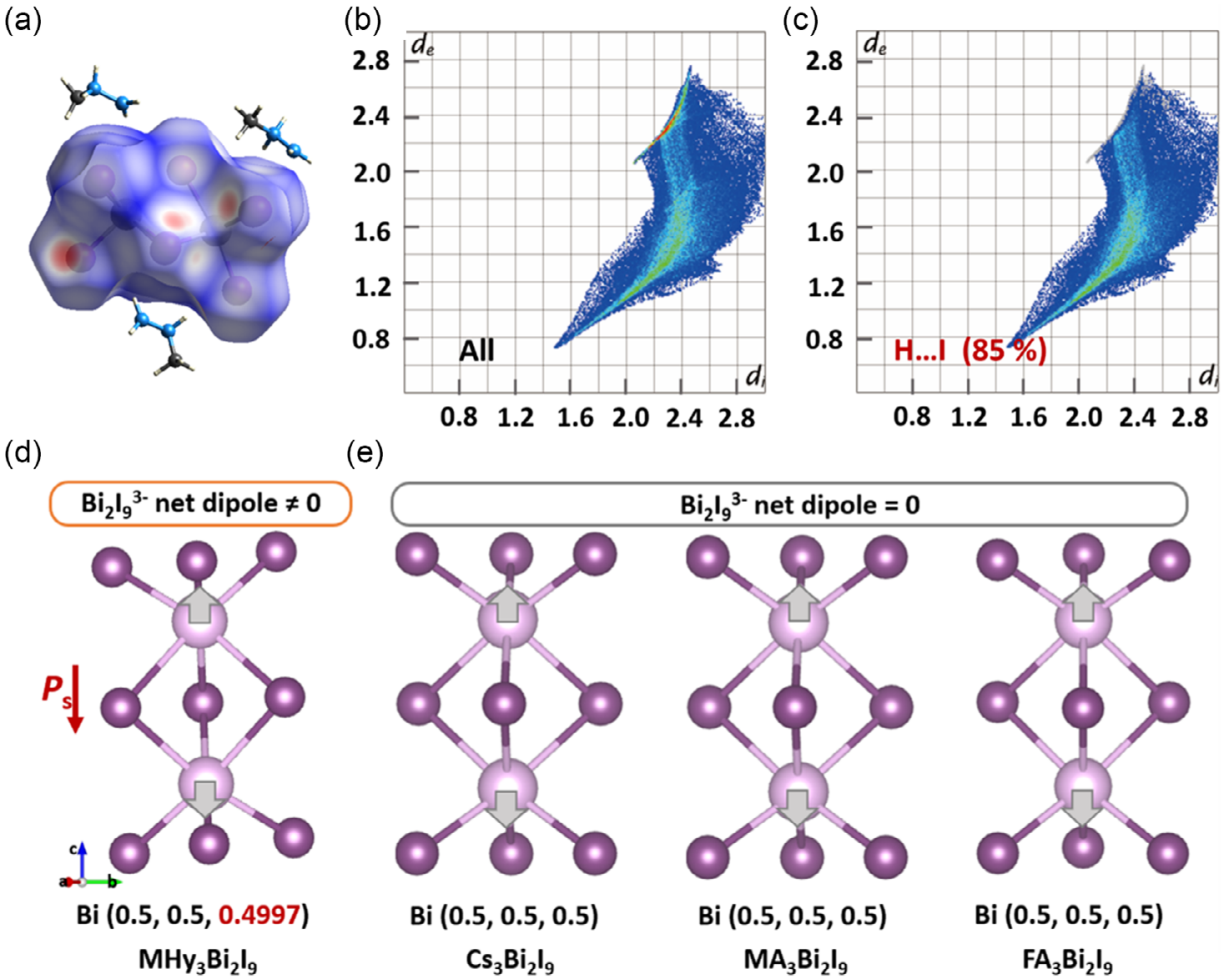
a) The Hirshfeld surface of the Bi_2_I_9_ unit in MHy_3_Bi_2_I_9_. b,c) The fingerprint plots of MHy_3_Bi_2_I_9_ indicate the strong hydrogen bond interaction. The inorganic unit of d) MHy_3_Bi_2_I_9_, and e) Cs_3_Bi_2_I_9_, MA_3_Bi_2_I_9_, and FA_3_Bi_2_I_9_. The gray arrows represent the direction of the BiI_6_ dipole moment. The red arrow in MHy_3_Bi_2_I_9_ refers to the direction of Bi_2_I_9_ net dipole moment, which points to the *c*‐axis.

In comparison with the centrosymmetric Cs_3_Bi_2_I_9_, MA_3_Bi_2_I_9_, and FA_3_Bi_2_I_9_, the chiral‐polar feature of MHy_3_Bi_2_I_9_ can be ascribed to the stereo‐active 2*s* ^2^ LPEs in MHy cation. Owning to the 2*s* ^2^ LPE located in the terminal *N* atom, the MHy cation forms the shorted hydrogen bond (2.31 Å). The hydrogen bond is much shorter than that of MA_3_Bi_2_I_9_ (2.74 Å), FA_3_Bi_2_I_9_ (2.69 Å), (Imidazolium)_3_Bi_2_I_9_ (2.86 Å), (aminoguanidine)_3_Bi_2_I_9_ (2.80 Å), and (benzylamine)_3_Bi_2_I_9_ (2.88 Å), which indicate the strong interaction between MHy cation and Bi_2_I_9_ inorganic framework (Figure 1f and Table S6, Supporting Information). We further analyze the Hirshfeld surface of the Bi_2_I_9_ unit (**Figure** **2**a). The fingerprint plots of MHy_3_Bi_2_I_9_ demonstrate the strong interaction effect between MHy organic cation and Bi_2_I_9_ inorganic dimer (Figure 2b,c). Second, due to the transfer of asymmetric properties of methylhydrazine to the inorganic skeleton, the two BiI_6_ dipole moment directions are not completely opposite, thus the Bi_2_I_9_ net dipole moment has deviated along the *c* direction, further contributing to the spontaneous polarization. For comparison, in the centrosymmetric A_3_Bi_2_I_9_‐type perovskites, the two BiI_6_ dipole moments in the Bi_2_I_9_ dimer point to an opposite direction, where the Bi_2_I_9_ net dipole moment is equal to zero (Figure S26, Supporting Information). Third, we calculated the coordinate to character the Bi atom's charge center displacement according to a point charge model.^[^ ^32^, ^33^ ^]^ As depicted in Figure 2d, the Bi atom coordinate charge center in MHy_3_Bi_2_I_9_ is (0.5, 0.5, and 0.4997), verifying the asymmetric Bi_2_I_9_ dimers, thus contributing to the formation of spontaneous polarization (Table S8, Supporting Information). In Cs_3_Bi_2_I_9_, MA_3_Bi_2_I_9_, and FA_3_Bi_2_I_9_, the Bi atoms’ off‐center displacements in a Bi_2_I_9_ dimer point to an opposite direction, and the Bi atom coordinate charge center is (0.5, 0.5, and 0.5), while the net dipole of the Bi_2_I_9_ dimer is equal to zero, consistent with the center symmetric *P*6_3_/*mmc* space group. These results demonstrate that owning to the 2*s* ^2^ LPEs, MHy is equipped with asymmetric properties and transmits the asymmetric properties to the Bi_2_I_9_ inorganic skeleton through hydrogen bonding, thus endowing the crystal structure with symmetry breaking.

#### 2.1.3 MHy Cation‐Induced Chiral‐Polar Characteristic

**Figure 3:**
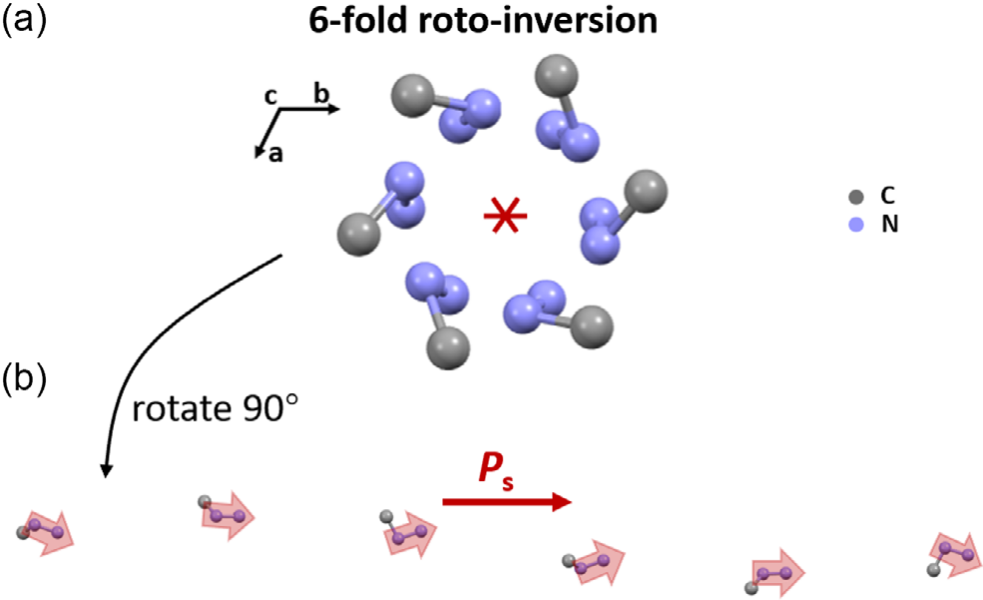
a) The 6‐fold roto‐inversion operation of MHy. *H* atoms are omitted for clarity. b) In the unit cell, the MHy dipole moment (light‐red arrow) contributes to the spontaneous polarization (*P*_s_, red arrow) along the c‐axis.

The dipole moments play an important role in spontaneous polarization. As shown in **Figure** **3**a,b, MHy dipole moment is arranged in a 6‐fold roto‐inversion along the *c*‐axis in the unit cell. Through the hydrogen bonding effect, spirally arranged MHy cation endows the Bi_2_I_9_ inorganic skeleton with spatial chirality. As shown in Figure S2, Supporting Information, the Bi_2_I_9_ dimer exhibits 2‐fold or 3‐fold roto‐inversion symmetry along the *c*‐axis, leading to MHy_3_Bi_2_I_9_'s chiral characteristic. Further, MHy_3_Bi_2_I_9_ SC discloses the obvious CD signal and unambiguously demonstrates chiroptical activity (Figure S3, Supporting Information). Meanwhile, the calculation verifies the MHy dipole moments and Bi_2_I_9_ dipole moments jointly contribute to MHy_3_Bi_2_I_9_'s polar characteristic (Figure S25 and Table S7, Supporting Information). In a word, MHy_3_Bi_2_I_9_ introduces a novel method to design polar perovskites by the spatially arranged MHy cation and promotes us to investigate the related polar characteristics and semiconductor properties.

### 2.2 Polarization Characteristics

**Figure 4:**
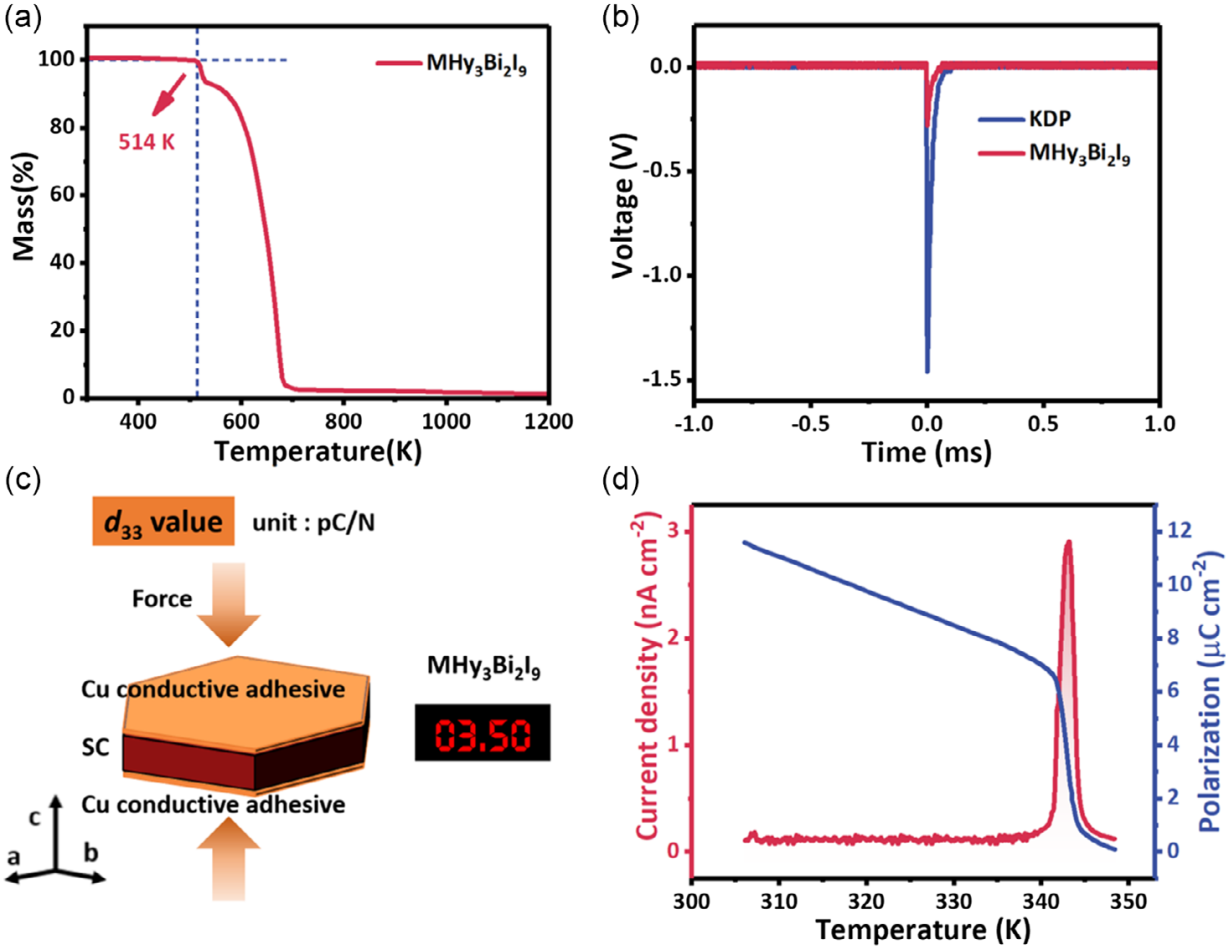
a) The thermogravimetric analysis experiment of MHy_3_Bi_2_I_9_. b) The second harmonic generation (SHG) test of MHy_3_Bi_2_I_9_ and KH_2_PO_4_. c) Piezoelectric measurement and the corresponding *d*_33_ value. d) The temperature‐dependent pyroelectricity current and polarization curves.

It is essential to perform the experimental evidence to illustrate the material's polarity. We carry the experimental powder X‐ray diffraction (PXRD), which matches the simulated PXRD pattern well and demonstrates the phase purity (Figure S4, Supporting Information). After being exposed to ambient air (relative humidity of 70%) for 30 days, the MHy_3_Bi_2_I_9_ PXRD patterns match well with the fresh sample, which discloses good air stability (Figure S5, Supporting Information). The thermogravimetric analysis displays a high thermostability in MHy_3_Bi_2_I_9_, which remains stable until 514 K and lays a solid foundation for the subsequent analysis (**Figure** **4**a). The second harmonic generation (SHG) test was performed with the KH_2_PO_4_ (KDP) as a reference. As shown in Figure 4b, MHy_3_Bi_2_I_9_ discloses an obvious SHG signal, indicating its non‐centrosymmetric feature. Because of the symmetry breaking, MHy_3_Bi_2_I_9_ single crystals (SCs) are naturally equipped with the piezoelectric response. As one of the direct piezoelectric coefficients, *d*_33_ indicates the perovskite's ability to generate an electric charge on a plane normal to the applied force. As depicted in Figure 4c, the Cu/MHy_3_Bi_2_I_9_ SC/Cu detector manifested a two‐terminal device with a bulk electrode structure, which was prepared with the Cu conductive adhesive parallel to the polar *c*‐axis, which exhibits a distinct *d*_33_ signal (3.5 pC N^−1^). The pyroelectric effect is an essential characteristic of polar materials and therefore an undoubted proof of the polar space group. The polarization exists in the polar space group while absent in the centrosymmetric space group. Therefore, the charge release can be seen near the phase transition temperature and manifests as an obvious and sharp pyroelectric peak (Figure 4d). The polarization value obtained by integrating the pyroelectric current is 11.6 μC cm^−2^, which coincides well with the polarization calculated from the point electric charge model (Figure S27 and Table S9, Supporting Information). Temperature‐dependent polarization vanishes above 343 K, indicating a polar–nonpolar phase transition. Single crystal X‐ray diffraction verifies the structure translates to the centrosymmetric *P*6_3_/*mmc* above 343 K (Table S3–S5, Supporting Information). Among the reversible phase transitions, the MHy^+^ organic cations suffer from the order–disorder transition (Figure S6, Supporting Information). In detail, the MHy^+^ organic cations are ordered at the RT phase and contribute to the structural chiral and polar features. As the temperature increases to the HT phase, the MHy^+^ organic cations are highly disordered, and the corresponding dipole moments are canceled out. Therefore, the order–disorder phase transition resulted in the charge release of around 343 K. The differential scanning calorimetry shows two thermal peaks at 343 and 334 K in the heating and cooling processes, demonstrating the reversible phase transition (Figure S7, Supporting Information). The temperature‐dependent PXRD patterns were performed to further verify the reversible phase transition (Figure S8, Supporting Information). The directional‐dependent pyroelectric and piezoelectric tests demonstrate the asymmetric characteristic properties of the obtained MHy_3_Bi_2_I_9_ SC (Figure S9, Supporting Information). The above measurement proves the polar characteristics of MHy_3_Bi_2_I_9_ and reveals the correlation between crystal structure and polarization.

### 2.3 X‐Ray Detection Characteristics

**Figure 5:**
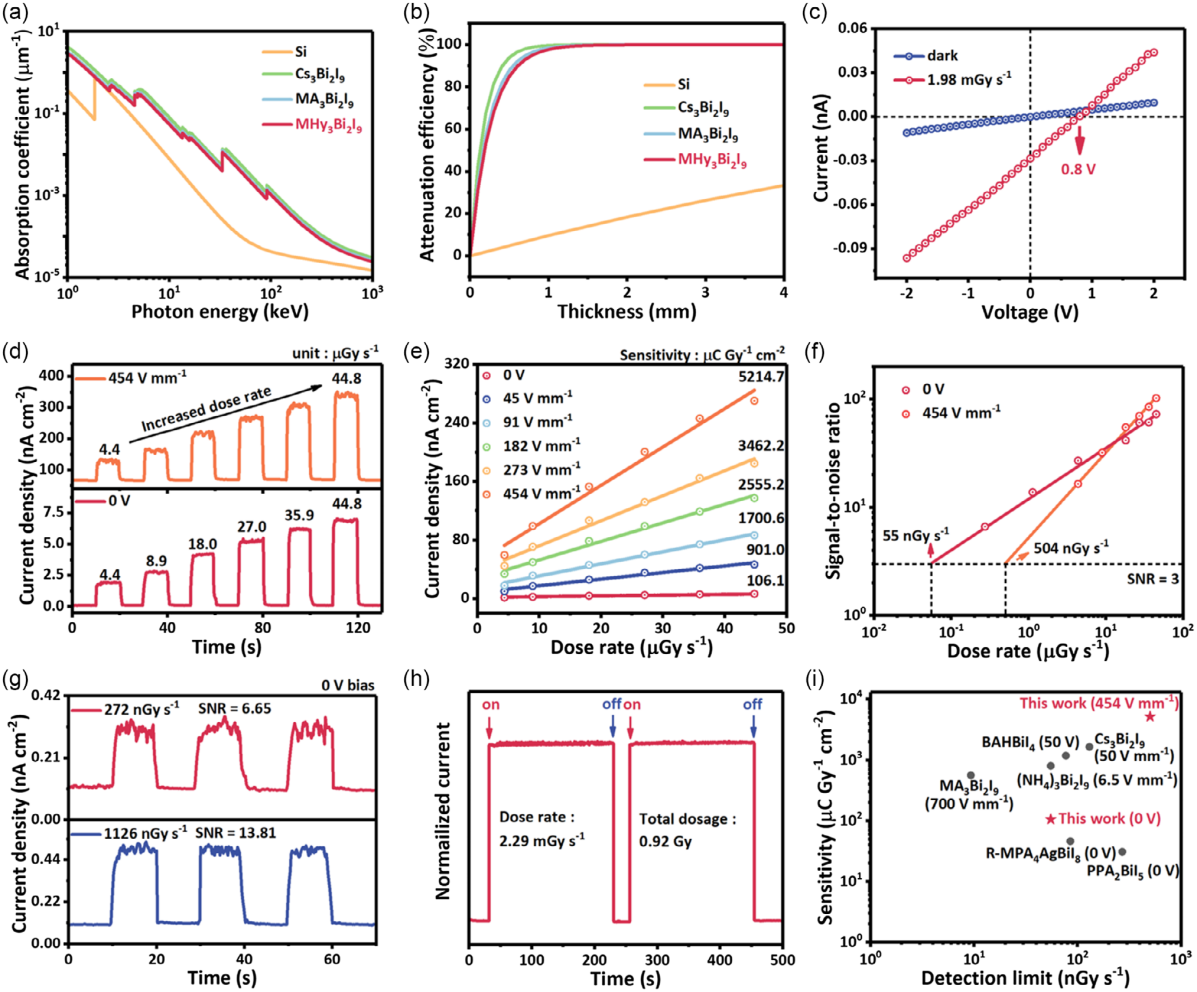
a) The absorption coefficient of MHy_3_Bi_2_I_9_, Cs_3_Bi_2_I_9_, MA_3_Bi_2_I_9_, and Si as a function of X‐ray photon energy. b) Attenuation efficiency versus thickness curves of MHy_3_Bi_2_I_9_, Cs_3_Bi_2_I_9_, MA_3_Bi_2_I_9_, and Si. c) *I*–*V* curves of MHy_3_Bi_2_I_9_ SC‐based detector in the dark and under X‐ray irradiation. d) The photo‐responses of the MHy_3_Bi_2_I_9_ SC‐based detector under increased X‐ray dose rate at 454 V mm^−1^ electric field (top) and 0 V bias (bottom). e) Under different external fields, the current density as a function of the X‐ray dose rate, and the slope of fitting lines corresponding to the sensitivity. f) The SNR of MHy_3_Bi_2_I_9_ SC‐based detector under 0 V and 454 V mm^−1^ electric field. g) The current response cycle of the detector under a low X‐ray dose rate to verify the reliability of the detection limit. h) The photocurrent of the detector under continuous X‐ray irradiation indicates its remarkable irradiation stability. i) The comparison of sensitivity and detection limit between MHy_3_Bi_2_I_9_ and other lead‐free detectors.

We further investigate the corresponding X‐ray detection properties that rely on the excellent spontaneous polarization characteristic. MHy_3_Bi_2_I_9_ SC manifests as a hexagon shape (Figure S10, Supporting Information). The crystal morphology was further analyzed according to the atomic force microscope (Figure S11, Supporting Information) and scanning electron microscope (Figure S12, Supporting Information) measurements, which disclose a flat and smooth crystal surface, verifying the high crystal quality. The light absorption measurements have been performed. The bandgap is calculated as 1.83 eV (Figure S13, Supporting Information). The valence band and conductive band positions were also analyzed according to the ultraviolet photoelectron spectroscopy (Figure S14, Supporting Information). The space charge limited current (SCLC) test discloses a low trap density of about 1.60 × 10^10^ cm^−3^, further verifying the crystal quality and low defect density (Figure S15, Supporting Information). Crystal with high crystallinity in a dimension of about 3.2 × 2.8 ×1 mm^3^ has been selected and coated with Cu conductive adhesive for X‐ray detection (Figure S16, Supporting Information). Exploiting the photon cross‐section database, the absorption coefficients of MHy_3_Bi_2_I_9_, Cs_3_Bi_2_I_9_, MA_3_Bi_2_I_9_, and Si were calculated to compare their X‐ray attenuation ability. As depicted in **Figure** **5**a, MHy_3_Bi_2_I_9_ exhibits a high absorption coefficient, which is comparable with the star compound Cs_3_Bi_2_I_9_, MA_3_Bi_2_I_9_, and is much higher than Si in a broad photon energy range (1–1000 keV). These materials’ attenuation efficiency versus thickness for 50 keV X‐ray photons was further calculated (Figure 5b). A MHy_3_Bi_2_I_9_ SC with 1 mm‐thick will absorb about 97.8% of incident photons, which is 10 times larger than that of Si (9.7%) and is on par with that of Cs_3_Bi_2_I_9_ (99.6%) and MA_3_Bi_2_I_9_ (98.5%). The bulk resistivity (*ρ*) is measured as 6.7 × 10^10^ Ω cm, which is similar to other 0D SC‐based detectors, such as Cs_3_Bi_2_I_9_ (2.8 × 10^10^ Ω cm), and AG_3_Bi_2_I_9_ (AG =aminoguanidinium, 2.4 × 10^10^ Ω cm).^[^ ^34^ ^]^ The large bulk resistivity can effectivity suppress the dark current and noise current, further allowing better X‐ray detection performance (Figure S17, Supporting Information). In addition, as a crucial X‐ray detection index, the efficient charge collection can be evaluated by mobility‐lifetime product (*μτ*). The *μτ* value can be extracted by fitting the current versus voltage curve of the MHy_3_Bi_2_I_9_ SC‐based detectors under X‐ray irradiation based on the following Hecht equation.

(1) $$I=\frac{I_{0}\mu \tau V}{L^{2}}\left[1-\exp\left(-\frac{L^{2}}{\mu \tau V}\right)\right]$$

where *I*, *I*_0_, *L*, and *V* refer to the photocurrent, saturated photocurrent, electrode spacing, and bias voltage, respectively. As depicted in Figure S18, Supporting Information, the *μτ* value is calculated as 6.26 × 10^−4^ cm^−2^ V^−1^, which is higher than some reported bismuth iodide perovskite SC‐based detectors, for instance, FA_3_Bi_2_I_9_ (2.4 × 10^−5^ cm^−2^ V^−1^) and (R‐1‐phenylpropylamine)_2_BiI_5_ (5.6 × 10^−5^ cm^−2^ V^−1^).^[^ ^35^ ^]^ Overall, the strong absorption coefficient, high bulk resistivity, and large *μτ* value make MHy_3_Bi_2_I_9_ SC a promising candidate for high‐performance X‐ray detection. As depicted in Figure 5c, MHy_3_Bi_2_I_9_ SC‐based detectors disclose a significant bulk photovoltaic effect (BPVE, 0.8 V) according to the *I*–*V* curves under X‐ray irradiation conditions. The detector discloses X‐ray‐induced currents because the electrode configuration is along the direction of spontaneous polarization (Figure S16, Supporting Information). On the one hand, BPVE enables the X‐ray‐generated carriers’ separation and transport, thus endowing the SC‐based detectors with X‐ray detection ability even at zero bias. On the other hand, under the self‐powered mode, the reduced dark current and noise current avail for a lower detection limit.

As shown in Figure 5d, the *I*–*t* curves display the temporal response of the MHy_3_Bi_2_I_9_ SC‐based detector toward the X‐ray irradiation. Benefitting from the BPVE, the detector exhibits a notable response to X‐ray even at 0 V external bias. The photocurrent density (*J*_ph_) reveals a linear increase from 1.93 nA cm^−2^ to 6.98 nA cm^−2^ with the X‐ray dose rate increasing from 4.4 to 44.8 μGy s^−1^, illustrating the excellent X‐ray response ability. Interestingly, BPVE in MHy_3_Bi_2_I_9_ SC shows directional dependency, which can be detected along the *c*‐axis while absence along the *a*‐axis and *b*‐axis, demonstrating the asymmetric self‐powered X‐ray detection properties (Figure S19, Supporting Information). For comparison, the photo response under different external fields (45, 91, 182, 273, and 454 V mm^−1^) was also performed (Figure S20–S23, Supporting Information). Due to the higher charge transfer efficiency with the increasing external bias, the current density increases sharply. For example, under the same X‐ray dose rate (44.8 μGy s^−1^), the photocurrent increased to 343.4 nA cm^−2^ at 454 V mm^−1^ external field, which is far larger than that at zero bias (6.98 nA cm^−2^). Meanwhile, as shown in Figure S15, Supporting Information, the dark current (*I*_dark_) of the MHy_3_Bi_2_I_9_ SC detector is 7 nA cm^−2^ @ 45 V mm^−1^, which is smaller than that of the MA_3_Bi_2_I_9_ SC‐based detector (10 nA cm^−2^ @ 40 V mm^−1^).^[^ ^10^ ^]^ It can be concluded that the large bulk resistivity and strong hydrogen bond interaction effectively reduce the dark current. As one of the important X‐ray detection indexes, sensitivity (*S*) represents the ability to collect charge per unit area under X‐ray irradiation and can be calculated by the formula.

(2) $$S=\left(J_{\text{ph}}-J_{\text{dark}}\right)/D$$

where *J*_ph_ is the current density under X‐ray irradiation, *J*_dark_ is the current density under dark condonation, and *D* is the X‐ray dose rate. As shown in Figure 5e, the current density (*J*_ph_ – *J*_dark_) is linearly corresponding to the dose rates. The sensitivity can be extracted by fitting their slopes, which are 106.1 μC Gy^−1^ cm^−2^ at 0 V and 5214.7 μC Gy^−1^ cm^−2^ at 454 V mm^−1^ external field. Overall, the lead‐free perovskite MHy_3_Bi_2_I_9_ displays a considerable X‐ray detection ability even compared with lead‐based perovskites.

The detection limit is another important parameter for X‐ray detection, which is defined as the dose rate with a SNR of 3. The SNR can be calculated from the formula

(3) $$\text{SNR}=\frac{I_{\text{signal}}}{I_{\text{noise}}}=\frac{\bar{I_{\text{photo}}}-\bar{I_{\text{dark}}}}{\sqrt{\frac{1}{n}\sum_{i}^{n}{\left(I_{i}-\bar{I_{\text{photo}}}\right)}^{2}}}$$

where $\bar{I_{\text{photo}}}$ is the average photocurrent, the $\bar{I_{\text{dark}}}$ is the average dark current, and *I*_noise_ is the noise current and can be acquired via calculating the standard deviation of the *I*_photo_. Based on the current versus time curves, the SNRs of different dose rates and different external voltages were calculated and displayed in Figure 5f and S20–S23, Supporting Information. Owning to the BPVE, the SNR of the MHy_3_Bi_2_I_9_ detector under 4.4 μGy s^−1^ is calculated as 27.1 even at zero bias, demonstrating the great potential toward weak X‐ray detection. By fitting the SNRs as a function of dose rate, the LoD can be obtained as 55 nGy s^−1^ at zero bias, which is 100 times lower than the regular medical diagnostics (5.5 μGy s^−1^). We further performed the X‐ray irradiation on‐off cycles under the lower dose rate and calculated the corresponding SNR. The SNR is calculated as 6.65 at a low X‐ray dose rate of 272 nGy s^−1^ and calculated as 13.81 at 1126 nGy s^−1^, indicating the detectors’ repeatability and reliability (Figure 5g). To further assess MHy_3_Bi_2_I_9_'s rad‐stability, the SC‐based detector was placed under an X‐ray generator to suffer the continued X‐ray irradiation of a large dose rate of 2.2 mGy s^−1^. Notably, the optical responsiveness remains favorable stability even when receiving a large dosage of up to 0.92 Gy, revealing excellent stability (Figure 5h). After being exposed to ambient air (relative humidity of 70%) for 30 days, the MHy_3_Bi_2_I_9_ SC‐based detector also disclosed a considerable X‐ray response, further verifying the self‐driven detection ability and air stability (Figure S24, Supporting Information). Notably, spontaneous polarization endows the MHy_3_Bi_2_I_9_ SC‐based detectors with self‐driven detection ability. Meanwhile, the noise current and dark current under zero bias have been suppressed, therefore acquiring a LoD of about 55 nGy s^−1^, which is lower than other A_3_Bi_2_I_9_‐type perovskites detectors, such as Cs_3_Bi_2_I_9_ (130 nGy s^−1^), MA_3_Bi_2_I_9_ (83 nGy s^−1^), and FA_3_Bi_2_I_9_ (200 nGy s^−1^), as depicted in Figure 5i and Table S10, Supporting Information. With the applied external field, the sensitivity of the MHy_3_Bi_2_I_9_ SC‐based detector increases to 5214.7 μC Gy^−1^ cm^−2^ @ 454 V mm^−1^, this value is higher than that of other star lead‐free perovskites, for example, Cs_3_Bi_2_I_9_ (*S* = 1652.3 μC Gy^−1^ cm^−2^ @ 50 V mm^−1^), MA_3_Bi_2_I_9_ (*S* = 563 μC Gy^−1^ cm^−2^ @ 700 V mm^−1^), and (NH_4_)_3_Bi_2_I_9_ (*S* = 803 μC Gy^−1^ cm^−2^ @ 6.5 V mm^−1^). Overall, benefitted from the strong absorption coefficient, high bulk resistivity, large *μτ* value, and significant bulk photovoltage, MHy_3_Bi_2_I_9_ exhibits superior self‐driven X‐ray detection ability.

## 3 Discussion

In summary, by exploiting the organic unit MHy with 2*s* ^2^ LPEs, we successfully obtained a chiral‐polar MHy_3_Bi_2_I_9_ in the famous A_3_Bi_2_I_9_‐type perovskite family. We systematically explain that the asymmetric MHy results in the asymmetric Bi_2_I_9_ dimer through hydrogen bond interaction, jointly contributing to the spontaneous polarization of MHy_3_Bi_2_I_9_. The polar characteristic was affirmed by the temperature‐dependent pyroelectric test. Importantly, the suppressed dark current and noise current under spontaneous polarization leads to an ultra LoD (55 nGy s^−1^) at zero bias, which is one percent of the conventional medical diagnostic dose. Our work demonstrates the first incorporation of LPEs organic cation into lead‐free perovskite and unprecedentedly performed self‐powered X‐ray detection in the famous A_3_Bi_2_X_9_‐type perovskites.

## Conflict of Interest

The authors declare no conflict of interest.

## Author Contributions

**Ruiqing Li**: Writing—original draft (lead); writing—review and editing (lead). **Jianbo Wu**: Writing—review and editing (supporting). **Zeng‐Kui Zhu**: Writing—review and editing (supporting). **Yaru Geng**: Writing—review and editing (supporting). **Xinling Li**: Writing—review and editing (supporting). **Yifei Wang**: Writing—review and editing (supporting). **Bohui Xu**: Writing—review and editing (supporting). **Zheshuai Lin**: Writing—review and editing (supporting). **Junhua Luo**: Writing—review and editing (lead).

## Supporting information

Supplementary Material

## Data Availability

The data that support the findings of this study are available from the corresponding author upon reasonable request.
