# Supplementary material for: Methylhydrazine Lone‐Pair Engineering for Polar Lead‐Free Perovskite Enables Self‐Powered X‐Ray Detection
Source: Small Sci. 2025 Mar 11;5(5):2400508. doi: 10.1002/smsc.202400508 (PMC12087766; doi:10.1002/smsc.202400508)
Supplement: Supplementary file 1 — Supplementary Material [file SMSC-5-2400508-s001.pdf]

Supporting Information

Methylhydrazine Lone-pair Engineering for Polar Lead-free Perovskite Enables Self-powered X-ray Detection

*Ruiqing Li, Jianbo Wu, Zeng-kui Zhu, Yaru Geng, Xinling Li, Yifei Wang, Bohui Xu, Zheshuai Lin, and Junhua Luo**

Experimental Details

Synthesis and crystal growth.

The $MHy_3Bi_2I_9$ were fabricated by dissolving stoichiometric Bi_2O_3 (16.8 mmol, 4g), $CH_3NHNH_2{\cdot}H_2SO_4$ (25 mmol, 2.9g) in HI solution (57%, 20 mL), particularly. H_3PO_2 (1 mL) was added to the solution to avoid the oxidization. Further, the mixed solution was heated to boiling under a constant magnetic stirring. After obtaining a clear solution, the heating and stirring stopped. Then the high-quality single crystals with large dimensions can be obtained in a slow cooling method (1 K/day) from 318 K to 288 K. The CCDC numbers are 2329620 and 2329619 for $MHy_3Bi_2I_9$ at room-temperature and high-temperature phases, respectively. The CCDC number is 2357317 for $FA_3Bi_2I_9$ at room temperature.

Circular dichroism (CD) measurement.

The solid-state CD spectroscopy of $MHy_3Bi_2I_9$ SC was performed on an MOS-450 spectrometer within a range of 220 − 820 nm at 298 K.

Powder X-ray diffraction (PXRD).

PXRD measurements were performed on a MiniFlex 600 instrument (Rigaku) with a Cu Ka radiation source. The diffraction patterns were collected in the 2θ range of 5° - 35° with a step size of 0.02°.

Thermogravimetric (TG) measurements.

The TG curve was conducted on a Netzsch STA 449C thermal analyzer with an N_2 flow rate of 30 ml/min and a heating rate of 15 K/min from 300 K to 1000 K.

The second harmonic generation (SHG) measurements.

The laser used for the SHG test is a Nd: YAG pulsed laser with a wavelength of 1064 nm, a peak power of 1.6MW, and a frequency of 10Hz. The particle size data for both $MHy_3Bi_2I_9$ and KH_2PO_4 are 270 μm.

Piezoelectric and pyroelectric measurements.

The single-crystal devices for piezoelectric and pyroelectric measurements were fabricated by depositing two symmetric Cu electrodes on the two opposite sides. The electrode materials were proven not to have any obvious influence on the photo-pyroelectric properties. The piezoelectric measurement was measured using a quasi-static d_{33} measuring instrument (ZJ-1AN). The temperature-dependent pyroelectric current was measured using an electrometer (Keithley 6517B) in a heating process with a constant heating rate.

X-ray detection.

A Keithley 6517B high-precision electrometer was used to record the current versus voltage (*I-V*) curves and current versus time (*I-t*) curves of $MHy_3Bi_2I_9$ SC devices. The X-ray source is an Amptek Mini-X2 X-ray tube with a silver target (maximum power 4 W). The maximum X-ray photon energy is 50 keV and the peak intensity is 22 keV. The dose rate of the X-ray tube was modulated by changing its tube current and measured by a Radcal Accu-Gold X-ray dosimeter attached to the 10 × 6 - 180 ion chamber in an integrating mode.

Calculation of electrical resistivity.

The electrical resistivity (ρ) was calculated based on the following formula,

$$\rho = \frac{U \times S}{A \times L}$$

where S, I, and L represent voltage, cross-sectional area, current, and material length, respectively.

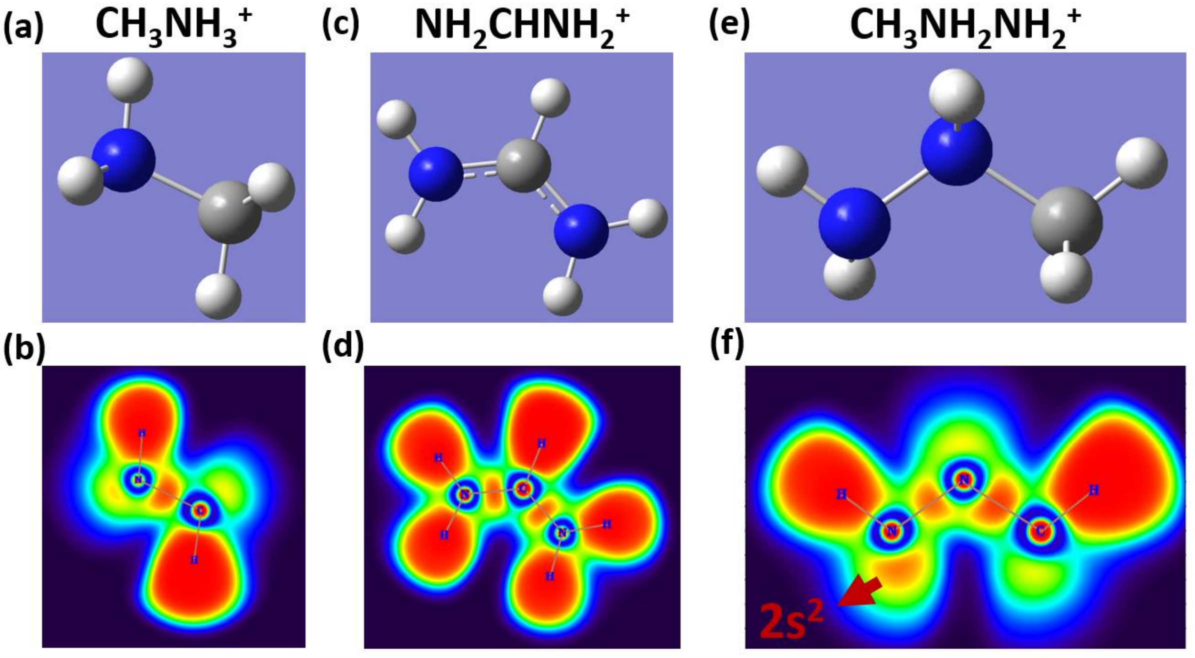

Figure S1. The ball-and-stick model of (a) $CH_3NH_3^+$ (c) $NH_2CHNH_2^+$ and (e) $CH_3NH_2NH_2^+$ organic cation and the corresponding electron localization function diagram of (b) $CH_3NH_3^+$ (d) $NH_2CHNH_2^+$ and (f) $CH_3NH_2NH_2^+$ organic cation. The orange crescent-shaped area in $CH_3NH_2NH_2^+$ refers to the stereo-active $2s^2$ lone-pair electrons of the terminal N atom.

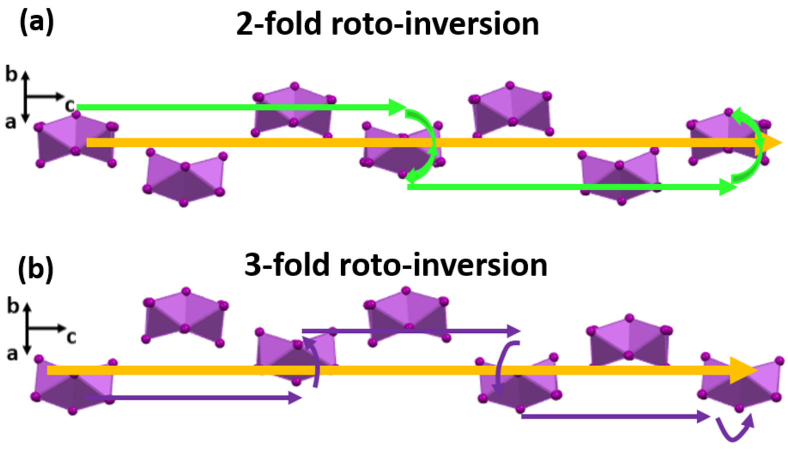

Figure S2. (a) The 2-fold roto-inversion operation of Bi_2I_9 dimer in $MHy_3Bi_2I_9$. (b) The 3-fold roto-inversion operation of Bi_2I_9 dimer in $MHy_3Bi_2I_9$.

The chiroptical activity of $MHy_3Bi_2I_9$ SC.

A bulk SC with a dimension of about 1*1*0.3 mm^3 was randomly selected, then ground into powder and pressed as wafers with KBr. As depicted in Figure S3, the obvious CD signal unambiguously demonstrates the chiroptical activity of the chiral-polar $MHy_3Bi_2I_9$ SC.

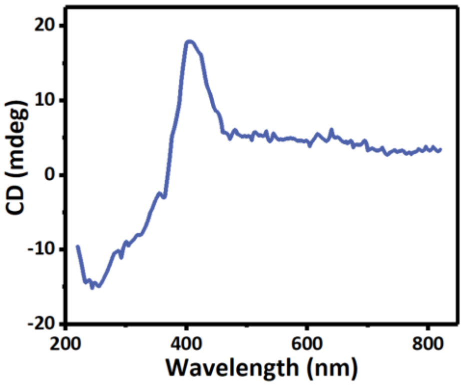

Figure S3. The circular dichroism (CD) spectrum of $MHy_3Bi_2I_9$ SC to verify the chiral feature.

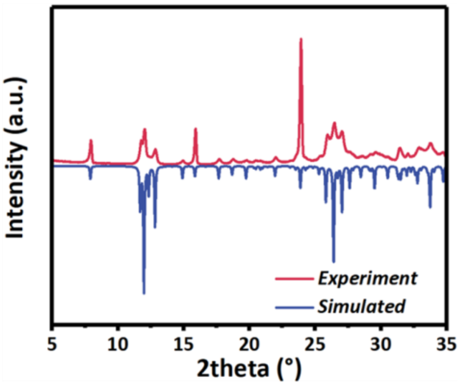

Figure S4. The powder X-ray diffraction (PXRD) pattern of $MHy_3Bi_2I_9$ to verify the pure phase.

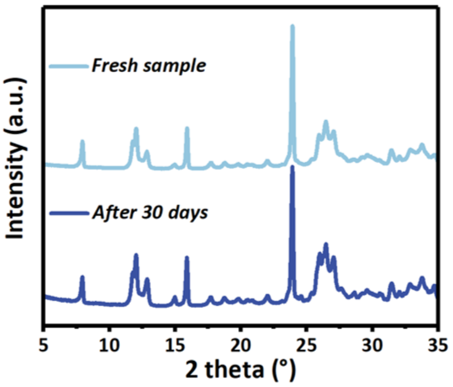

Figure S5. The PXRD comparison between the fresh sample and the sample exposed to ambient air (relative humidity of 70%) for 30 days to confirm the phasic stability.

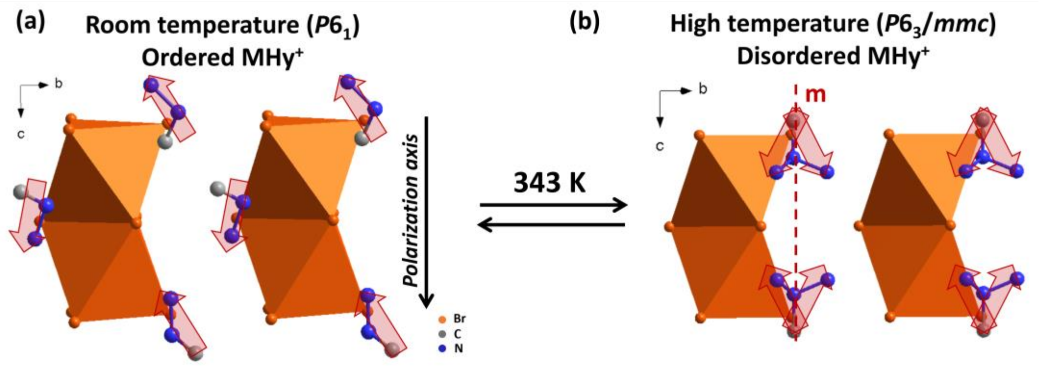

Figure S6. The crystal structure of $MHy_3Bi_2I_9$ at (a) low-temperature phase (301 K, $P6_1$) and (b) high-temperature phase (376 K, $P6_3/mmc$).

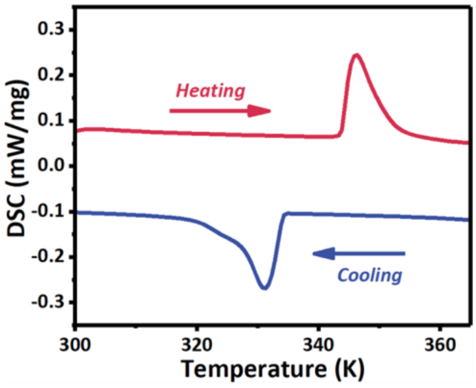

Figure S7. The DSC trace of $MHy_3Bi_2I_9$ under the heating and cooling process.

Temperature-dependent PXRD.

The temperature-dependent PXRD was tested from room temperature (RT), 323 K, 343 K, to 363 K, and finally reduced to RT, as shown in Figure S8.

Firstly, the PXRD at room temperature and 323 K match well with the simulated RT-PXRD patterns, indicating the pure phase. As the temperature increases to 343 K, the PXRD curve displays both the RT diffraction peaks and HT diffraction peaks, indicating it suffers from a phase transition. In detail, the HT diffraction peaks of (102) and (103) crystal planes gradually appear, while the RT diffraction peak of (104) (107) (1010) (1011) (1012) crystal peaks still exist. When the temperature further increases to 363 K, the RT diffraction peaks of (107) (1010) (1011) (1012) crystal planes disappear, and the corresponding PXRD curve matches well with simulated HT phase ($P6_3/mmc$), verifying $MHy_3Bi_2I_9$ transform into nonpolar HT phase from the polar RT phase. Finally, when the temperature reduces to RT, the diffraction peaks coincide well with the original RT and simulated RT curves, disclosing the reversible phase transition.

In a word, the temperature-dependent PXRD patterns verified the polar ($P6_1$) to nonpolar ($P6_3/mmc$) phase transition, which coincided with the SC X-ray diffractions and temperature-dependent polarization.

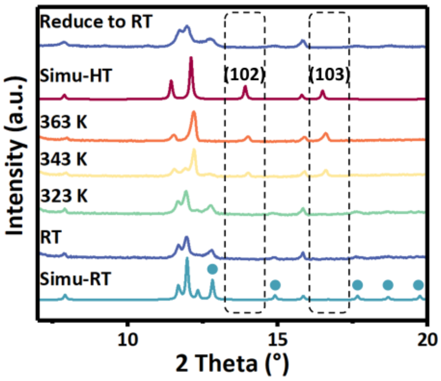

Figure S8. The temperature-dependent PXRD curves of $MHy_3Bi_2I_9$. The green dots refer to the simulated RT (104) (107) (10<u>10</u>) (10<u>11</u>) (10<u>12</u>) diffraction peaks, from left to right, respectively.

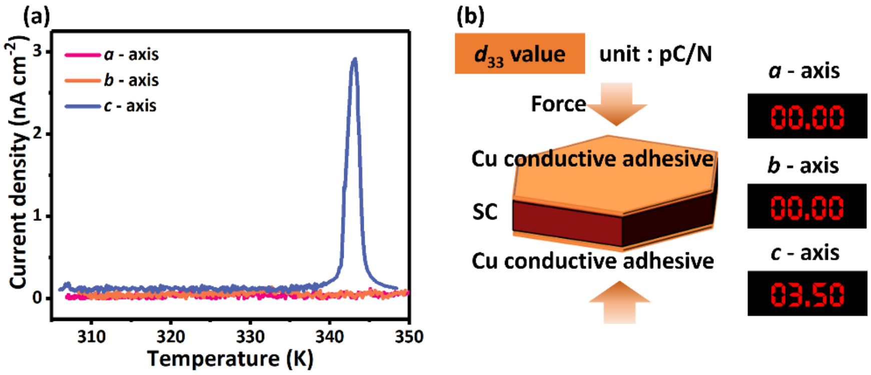

Figure S9. (a) The pyroelectric measurement and (b) piezoelectric measurement of $MHy_3Bi_2I_9$ SC along with different crystal orientations.

The analysis of $MHy_3Bi_2I_9$ SC and $MHy_3Bi_2I_9$ SC-based detector.

$MHy_3Bi_2I_9$ SC (Figure S9a) manifests as a hexagon shape, which matches well with the simulated morphology (Figure S9b). Further, the X-ray diffraction of the top facet of $MHy_3Bi_2I_9$ SC discloses periodic diffraction peaks along the (00*l*) direction, verifying the good crystal growth orientation (Figure S9c). Further, the schematic diagram and picture of the $MHy_3Bi_2I_9$ SC-based detector are provided in Figure S10a and Figure S10b, respectively. The dimension of single-crystal size is 3.2 × 2.8 × 1 mm^3. The Cu conductive adhesive was coated on the $MHy_3Bi_2I_9$ SC surface to ensure charge collection. The effective device area is 0.177 mm^2 and the electrode spacing is 0.11 mm.

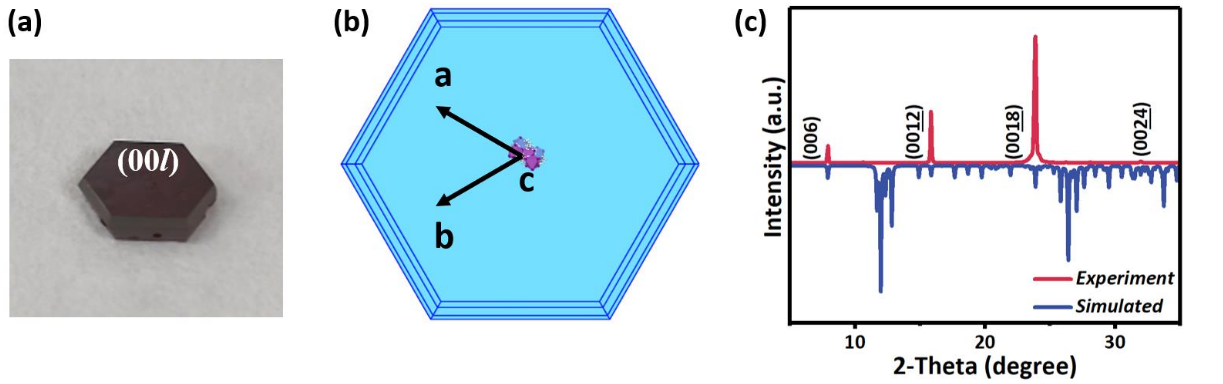

Figure S10. (a) The photograph of $MHy_3Bi_2I_9$ SC. (b) The simulated morphology of $MHy_3Bi_2I_9$. (c) The X-ray diffraction of $MHy_3Bi_2I_9$ SC wafer.

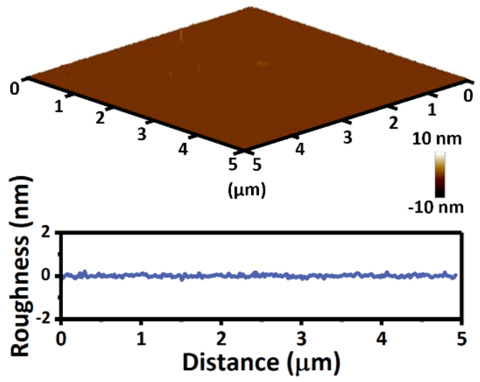

Figure S11. The atomic force microscope image and corresponding roughness of $MHy_3Bi_2I_9$ SC.

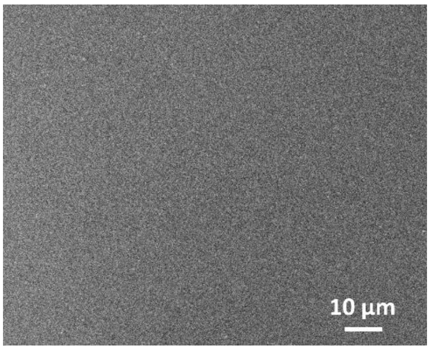

Figure S12. The scanning electron microscope image of $MHy_3Bi_2I_9$ SC.

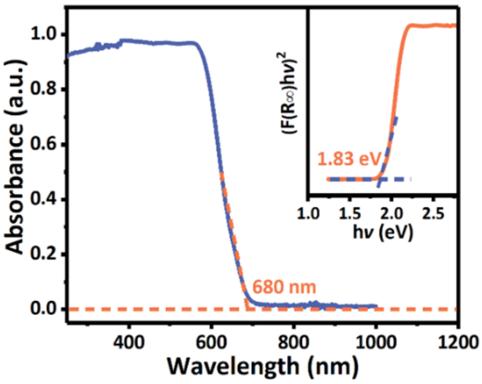

Figure S13. Ultraviolet-visible light absorption spectrum of $MHy_3Bi_2I_9$. Inset: the calculated bandgap.

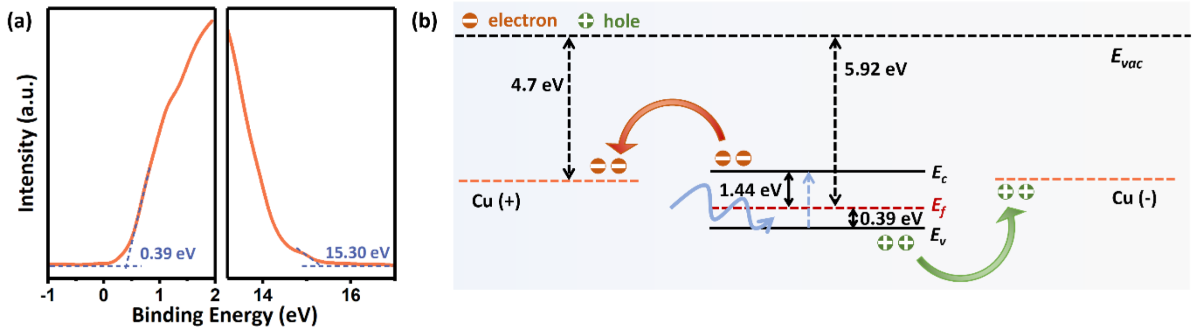

Figure S14. (a) The UPS measurement of $MHy_3Bi_2I_9$. (b) Schematic diagram of Fermi level position and bandgap edge based on the UPS and E_g. Where E_{vac}, E_c, E_v, and E_f refer to vacuum level, conduction band level, valence band level, and Fermi level, respectively.

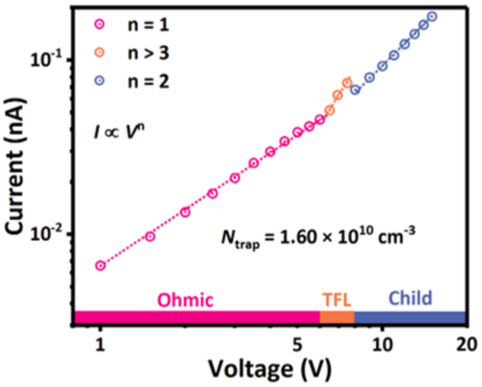

Figure S15. *I*-*V* measurements according to the SCLC method.

The device structure and corresponding bulk photovoltaic effect.

In the crystal structure of $MHy_3Bi_2I_9$, MHy^+ dipole moments and $Bi_2I_9^{3-}$ dipole moments contribute to the spontaneous polarization in the *c*-axis. The spontaneous polarization acts as a driving force to realize the X-ray-generated carriers' separation and transport to acquire a self-

powered detection ability, that is, bulk photovoltaic effect. (Please see: *Science*, 2009, *324*, 63-66; *Matter*, 2022, *5*, 9, 2659-2684.) As for the obtained SC, the polarization direction (*c*-axis) is perpendicular to the hexagonal crystal plane (Figures S16a and S16b). For the device structure in Figure S16c, the current direction is along the spontaneous polarization direction according to the electrode configuration. Therefore, the SC detector discloses an obvious open-circuit voltage and short-circuit current under X-ray irradiation (Figure 5c).

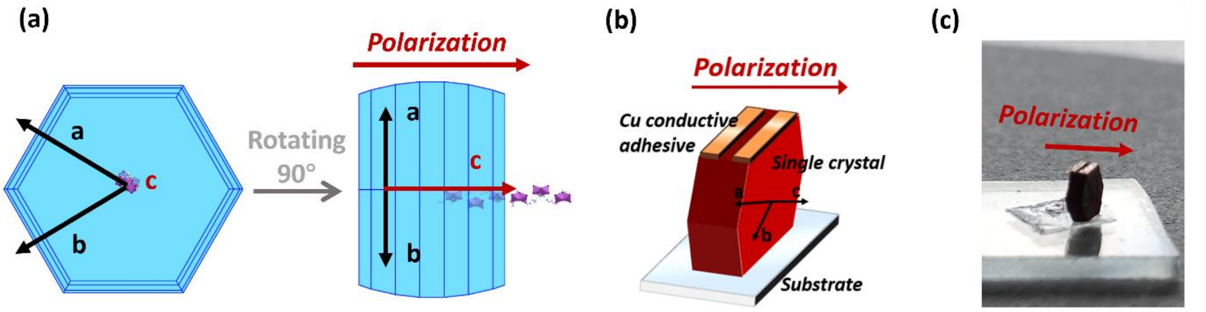

Figure S16. (a) The simulated SC morphology. The red arrow refers to the direction of polarization. (b) The schematic diagram of $MHy_3Bi_2I_9$ SC-based detector. (c) The picture of $MHy_3Bi_2I_9$ SC-based detector.

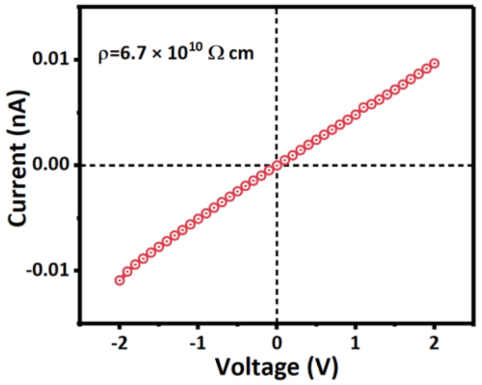

Figure S17. The *I*-*V* curve of $MHy_3Bi_2I_9$ SC and corresponding electrical resistivity.

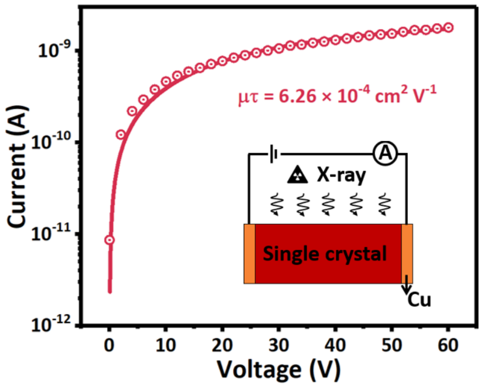

Figure S18. The *I*-*V* curve and calculated mobility-lifetime product.

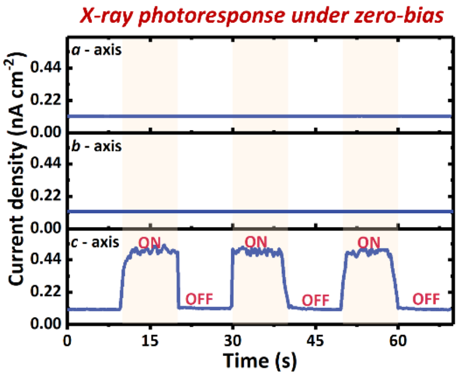

Figure S19. Under zero bias, the current density of $MHy_3Bi_2I_9$ SC-based detector along the *a*, *b*, and *c*-axes, respectively (X-ray dose rate: 1.12 μGy s^{-1})

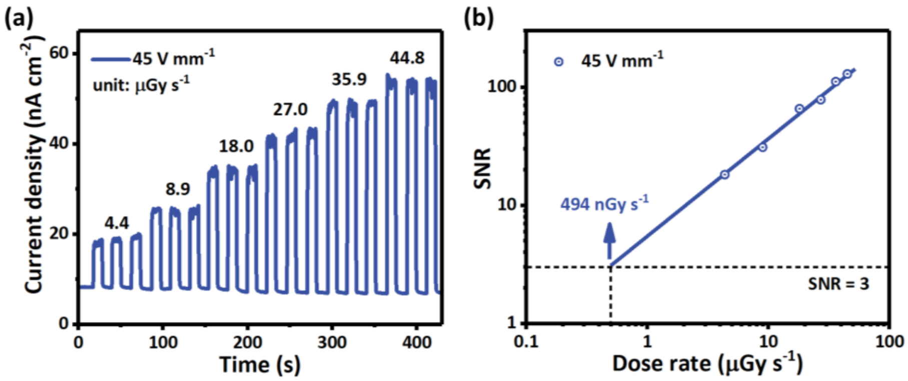

Figure S20. (a) The current density versus time curves of $MHy_3Bi_2I_9$ single crystal (SC) based detectors under increased X-ray dose rate. (b) SNR of $MHy_3Bi_2I_9$ SC-based detectors at 45 V mm^{-1} electric field, the low detection limit is 494 nGy s^{-1}.

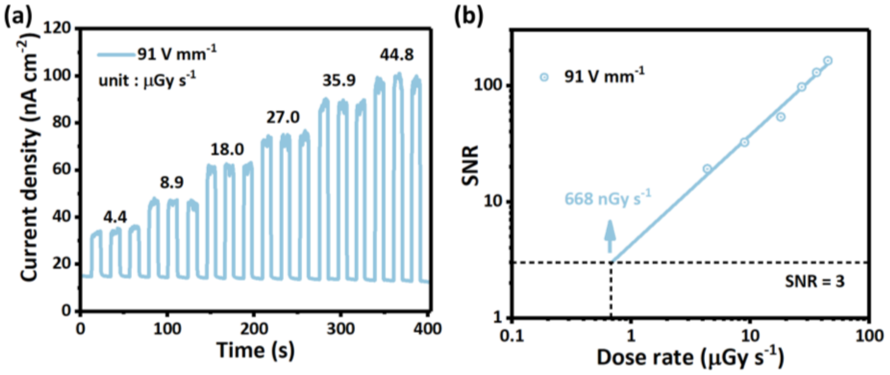

Figure S21. (a) The current density versus time curves of $MHy_3Bi_2I_9$ SC-based detectors under increased X-ray dose rate. (b) SNR of $MHy_3Bi_2I_9$ SC-based detectors at 91 V mm^{-1} electric field, the low detection limit is 668 nGy s^{-1}.

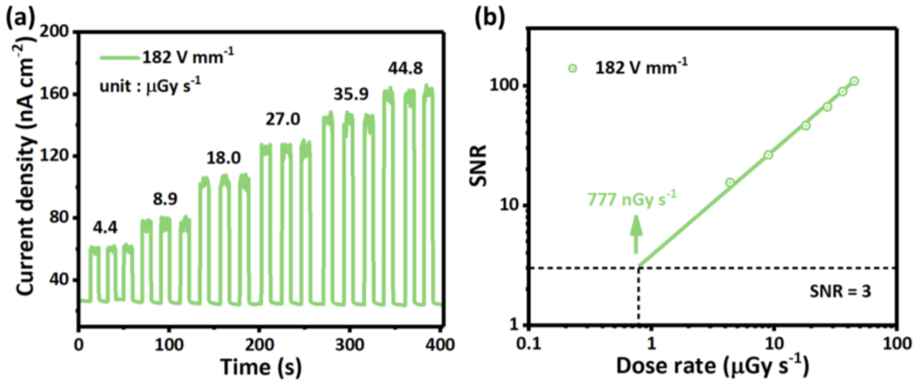

Figure S22. (a) The current density versus time curves of $MHy_3Bi_2I_9$ SC-based detectors under increased X-ray dose rate. (b) SNR of $MHy_3Bi_2I_9$ SC-based detectors at 182 V mm^{-1} electric field, the low detection limit is 777 nGy s^{-1}.

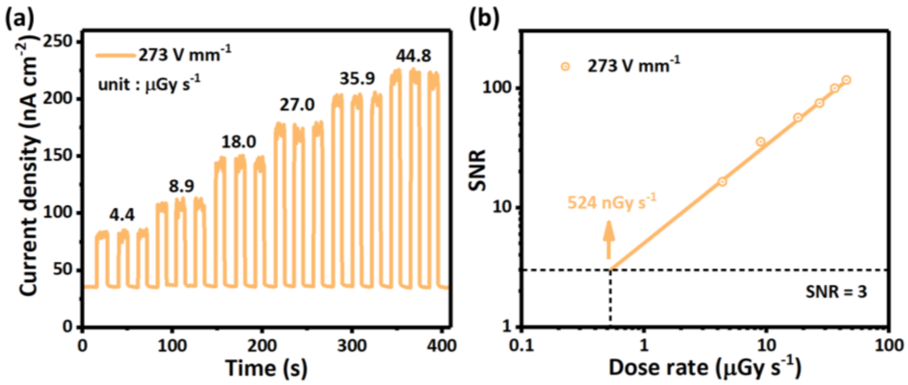

Figure S23. (a) The current density versus time curves of $MHy_3Bi_2I_9$ SC-based detectors under increased X-ray dose rate. (b) SNR of $MHy_3Bi_2I_9$ SC-based detectors at 273 V mm^{-1} electric field, the low detection limit is 524 nGy s^{-1}.

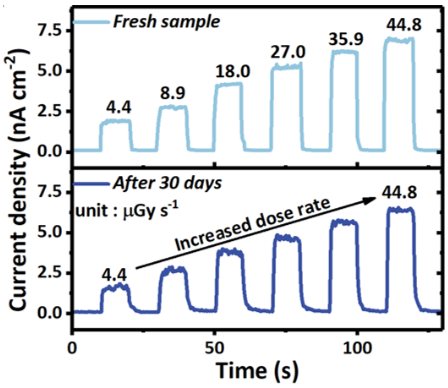

Figure S24. Under self-driven mode, the X-ray photoresponse of fresh sample (up) and sample after 30 days (down).

Table S1. Crystal data and structure refinement for $MHy_3Bi_2I_9$ at low-temperature (301 K) and high-temperature (376 K) phase.

Identification code	$(CH_3NH_2NH_2)_3Bi_2I_9$	
Temperature/K	301 K	376 K
Space group	$P6_1$	$P6_3/mmc$
a/Å	8.7402(11)	8.9194(6)
b/Å	8.7402(11)	8.9194(6)
c/Å	67.038(8)	22.414(2)
α/°	90	90
β/°	90	90
γ/°	120	120
Volume/Å^3	4435.0(12)	1544.3(3)
Z	6	2
ρ_{calc} g/cm^3	3.822	3.629
F(000)	4344.0	1414.0
Radiation	MoKα (λ = 0.71073)	MoKα (λ = 0.71073)
2Θ	5.382 to 49.994	7.272 to 57.152
Index ranges	-10≤h≤ 7, -10≤k≤10, -79≤l≤ 79	-11≤h≤9, -11≤k≤ 11, -27≤l≤29
Reflections collected	24246	13761
Independent reflections	5218 [R_{int} = 0.1034, R_{sigma} = 0.0869]	773 [R_{int} = 0.0845, R_{sigma} = 0.0484]
Data/restraints/parameters	5218/61/184	773/3/22
Goodness-of-fit on F^2	1.033	1.039
Final R indexes [I>=2σ(I)]	R_1 = 0.0583, wR_2 = 0.1323	R_1 = 0.0900, wR_2 = 0.2614
Final R indexes [all data]	R_1 = 0.1030, wR_2 = 0.1537	R_1 = 0.1519, wR_2 = 0.3232
Largest diff. peak/hole / e Å^{-3}	1.41/-1.45	1.74/-1.65

Table S2. Selected I-Bi-I bond angles of $MHy_3Bi_2I_9$ at low-temperature (301 K).

Atom	Atom	Atom	Angle/°	Atom	Atom	Atom	Angle/°
I5	Bi1	I6	81.68(10)	I7	Bi2	I5	94.21(10)
I3	Bi1	I5	94.08(11)	I7	Bi2	I6	93.88(10)
I3	Bi1	I6	174.46(12)	I7	Bi2	I4	174.42(12)
I3	Bi1	I4	93.99(9)	I7	Bi2	I9	93.15(12)
I3	Bi1	I1	93.06(12)	I7	Bi2	I8	92.25(12)
I3	Bi1	I2	92.44(12)	I9	Bi2	I5	89.70(12)
I4	Bi1	I5	82.74(10)	I9	Bi2	I6	170.06(13)
I4	Bi1	I6	81.98(9)	I9	Bi2	I4	90.48(11)
I1	Bi1	I5	89.75(12)	I8	Bi2	I5	171.27(13)
I1	Bi1	I6	90.49(11)	I8	Bi2	I6	90.95(13)
I1	Bi1	I4	170.04(12)	I8	Bi2	I4	91.60(10)
I2	Bi1	I5	171.13(13)	I8	Bi2	I9	95.80(16)
I2	Bi1	I6	91.43(11)	Bi1	I5	Bi2	81.43(5)
I2	Bi1	I4	90.81(13)	Bi2	I6	Bi1	81.23(7)
I2	Bi1	I1	95.91(15)	Bi1	I4	Bi2	81.36(7)
I5	Bi2	I4	81.55(9)	C2	N3	N4	111(4)
I6	Bi2	I5	82.77(10)	N2	N1	C1	101(3)
I6	Bi2	I4	82.02(9)	C3	N5	N6	112(4)

Table S3. Bi-I bond distances in the unit cell for $MHy_3Bi_2I_9$ at low-temperature (301 K).

Atom	Atom	Length/Å	Atom	Atom	Length/Å
Bi[1]	I5	3.245(3)	Bi[2]	I5	3.249(3)
Bi[1]	I6	3.291(3)	Bi[2]	I6	3.216(3)
Bi[1]	I3	2.936(3)	Bi[2]	I4	3.286(3)
Bi[1]	I4	3.213(3)	Bi[2]	I7	2.939(3)
Bi[1]	I1	2.966(3)	Bi[2]	I9	2.966(4)
Bi[1]	I2	2.945(4)	Bi[2]	I8	2.946(4)

Table S4. Bi-I bond distances in the unit cell for $MHy_3Bi_2I_9$ at high-temperature (376 K).

Atom	Atom	Length/Å	Atom	Atom	Length/Å
Bi1	I1[1]	3.263(2)	Bi1	I2[2]	2.956(3)
Bi1	I1	3.263(2)	Bi1	I2[1]	2.956(3)
Bi1	I1[2]	3.263(2)	Bi1	I2	2.956(3)

Table S5. Selected I-Bi-I bond angles of $MHy_3Bi_2I_9$ at high-temperature (376 K).

Atom	Atom	Atom	Angle/°	Atom	Atom	Atom	Angle/°
$I1^1$	Bi1	I1	82.30(6)	$I2^1$	Bi1	$I1^2$	91.94(7)
$I1^2$	Bi1	$I1^1$	82.30(6)	I2	Bi1	$I2^2$	93.33(13)
$I1^2$	Bi1	I1	82.30(6)	$I2^1$	Bi1	$I2^2$	93.33(13)
$I2^2$	Bi1	I1	91.94(7)	$I2^1$	Bi1	I2	93.33(13)
$I2^1$	Bi1	I1	172.33(10)	Bi1	I1	$Bi1^3$	81.10(8)
I2	Bi1	$I1^1$	91.94(7)	N2	N1	C1	113(3)
$I2^2$	Bi1	$I1^2$	91.94(8)	$N2^4$	N1	C1	113(3)
$I2^1$	Bi1	$I1^1$	91.94(7)	$N2^5$	N1	C1	113(3)
$I2^2$	Bi1	$I1^1$	172.33(10)	N2	N1	$N2^4$	106(4)
I2	Bi1	I1	91.93(7)	$N2^5$	N1	$N2^4$	106(4)
I2	Bi1	$I1^2$	172.32(10)	N2	N1	$N2^5$	106(3)

Table S6. The comparison of hydrogen bond effect between different $A_3Bi_2I_9$-type perovskites.

A-cation	effective radii (pm)	compound	hydrogen bond (Å)	Ref.
methylamine	217	$MA_3Bi_2I_9$	2.74	*Inorg. Chem.* 2017, *56*, 1, 33–41
formamidine	253	$FA_3Bi_2I_9$	2.69	*J. Mater. Chem. C*, 2019,*7*, 3003-3014
Imidazolium	258	$IM_3Bi_2I_9$	2.86	*Inorg. Chem. Front.* 2016,*3*, 1306-1316
**methylhydrazine**	**264**	**$MHy_3Bi_2I_9$**	**2.31**	**This work**
aminoguanidine	-	$AG_3Bi_2I_9$	2.80	*Adv. Mater.* 2023, *35*, 2211977
benzylamine	-	$BZA_3Bi_2I_9$	2.88	*Chem. Mater.* 2020, *32*, 6, 2647-2652

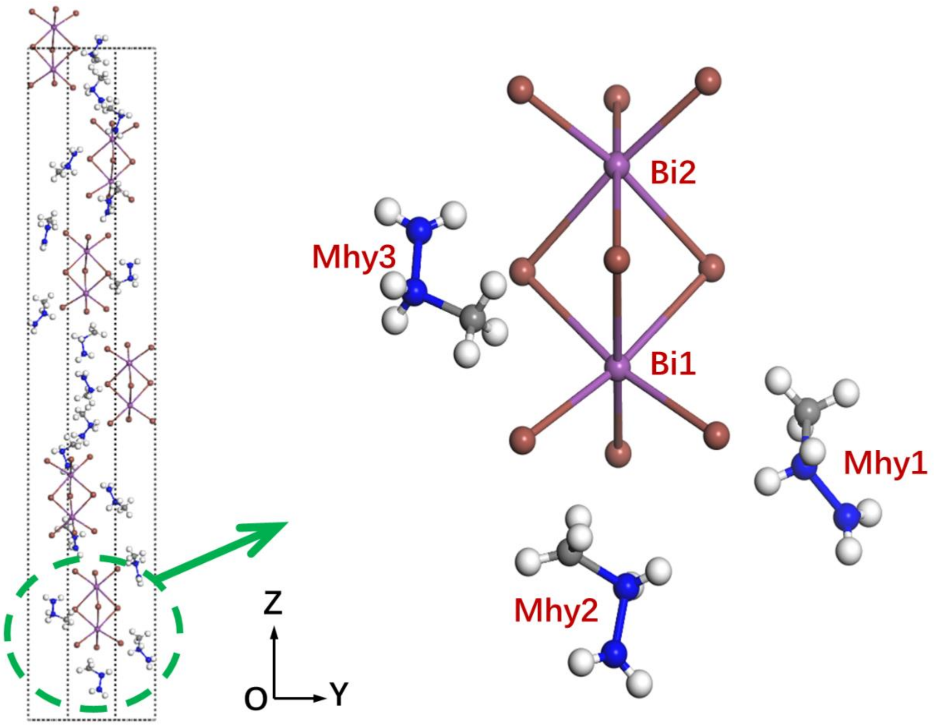

Figure S25. The unit cell of **$MHy_3Bi_2I_9$.**

Table S7. The calculated point charge model of **$MHy_3Bi_2I_9$.**

Species	Dipole			Magnitude
	x	y	z	
$[BiI_6]^1$	0.5601	0.0471	5.3947	5.4239
$[BiI_6]^2$	-0.2920	-0.4753	-5.4458	5.4742
$[Bi_2I_9]$	0.2681	-0.4282	-0.0511	0.5078
MHy^1	1.2706	-0.9770	-0.0611	1.6040
MHy^2	-0.7702	1.3109	0.5972	1.6335
MHy^3	0.7596	-1.2493	-0.6580	1.6033
Tot_{Organ}	1.2601	-0.9154	-0.1218	1.5622
Tot_{unit}	1.5281	-1.3436	-0.1729	2.0422
Tot_{cell}	0.0000	0.0000	-1.0373	1.0373

The comparison of Bi-I bond length between $MHy_3Bi_2I_9$ and $Cs_3Bi_2I_9$, $MA_3Bi_2I_9$, $FA_3Bi_2I_9$. Owning to the asymmetric organic cation MHy^+ and hydrogen-bond interaction, the asymmetric property transfer to the inorganic skeleton, results in six unequal Bi-I bonds (from 2.94 Å to 3.29 Å), as shown in Figure S26a. For comparison, in the centrosymmetric $Cs_3Bi_2I_9$ (Figure S26b), the Bi_2I_9 dimer has three equal long Bi-I bonds (3.24 Å) and three equal short Bi-I bonds (2.93 Å). A similar phenomenon can also be seen in $MA_3Bi_2I_9$ (Figure S26c), and $FA_3Bi_2I_9$ (Figure S26d). It is known that the *ns*^{2} lone-pair electrons of metal ions (Ge^{2+}, Sn^{2+}, Pb^{2+}, Sb^{3+}, Bi^{3+}) are always located on the side of long M-X bond (M = metal ion, X = halide ion). In this case, the two BiI_6 dipole moments in a Bi_2I_9 dimer point to an opposite direction in $Cs_3Bi_2I_9$, $MA_3Bi_2I_9$, and $FA_3Bi_2I_9$. In contrast, in $MHy_3Bi_2I_9$, owning the asymmetric organic cation and hydrogen-bond interaction, the two BiI_6 dipole moment directions are not completely opposite and result in a net dipole moment along the *c*-axis.

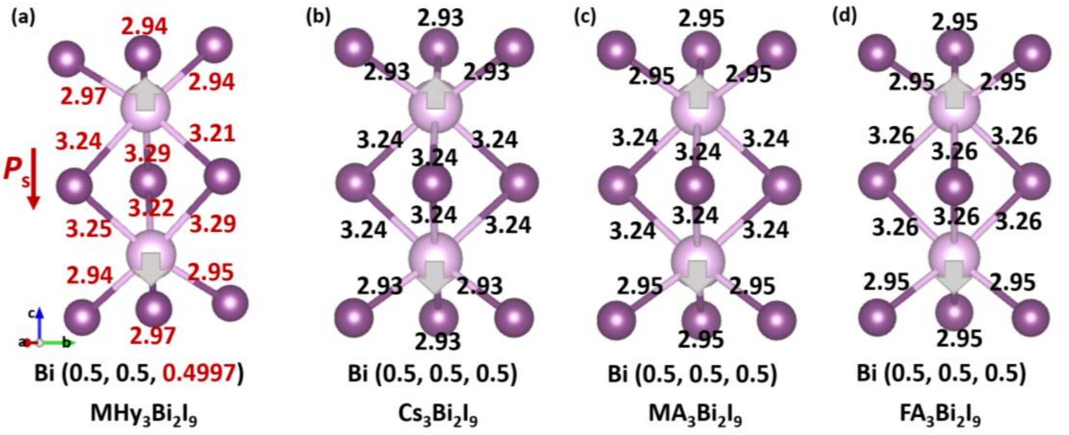

Figure S26. The Bi-I bond lengths in (a) $MHy_3Bi_2I_9$ (b) $Cs_3Bi_2I_9$ (c) $MA_3Bi_2I_9$ (d) $FA_3Bi_2I_9$.

The point charge model of $MHy_3Bi_2I_9$, $Cs_3Bi_2I_9$, $MA_3Bi_2I_9$, and $FA_3Bi_2I_9$.

The point charge model of $MHy_3Bi_2I_9$, $Cs_3Bi_2I_9$, $MA_3Bi_2I_9$, and $FA_3Bi_2I_9$ were calculated to further verify the $MHy_3Bi_2I_9$'s net dipole moment along the *c*-axis. As depicted in Table S7, the Bi atoms' coordinate charge centers in $Cs_3Bi_2I_9$ are (0.5,0.5,0.5956) and (0.5,0.5,0.4044), while the coordinate charge center of Bi_2I_9 dimer is (0.5, 0.5, 0.5), indicating the Bi atoms' off-center displacement in $Cs_3Bi_2I_9$ point the two completely opposite directions and counterbalanced. In contrast, the coordinate charge center of Bi_2I_9 dimer in $MHy_3Bi_2I_9$ is (0.5, 0.5, 0.4997), showing that the dipole moments are not completely canceled due to the presence of hydrogen bonds and asymmetric organic cation MHy. The net dipole moment of Bi_2I_9 dimer in $MHy_3Bi_2I_9$ points to the polarization direction: *c*-axis, coinciding with the $P6_1$ spatial symmetry.

Table S8. The comparison of Bi atom coordination charge center between $MHy_3Bi_2I_9$, $Cs_3Bi_2I_9$, $MA_3Bi_2I_9$, and $FA_3Bi_2I_9$.

Compound	Coordinate charge center		
	Bi1	Bi2	$Total_{inor}$
$MHy_3Bi_2I_9$	(0.5, 0.5, 0.44805)	(0.5, 0.5, 0.5515)	(0.5, 0.5, **0.4997**)
$Cs_3Bi_2I_9$	(0.5, 0.5, 0.5956)	(0.5, 0.5, 0.4044)	(0.5, 0.5, 0.5)
$MA_3Bi_2I_9$	(0.5, 0.5, 0.4091)	(0.5, 0.5, 0.5949)	(0.5, 0.5, 0.5)
$FA_3Bi_2I_9$	(0.5, 0.5, 0.59498)	(0.5, 0.5, 0.40502)	(0.5, 0.5, 0.5)

Calculation of polarization value (*Ps*) of **$MHy_3Bi_2I_9$** according to a point electric charge model.

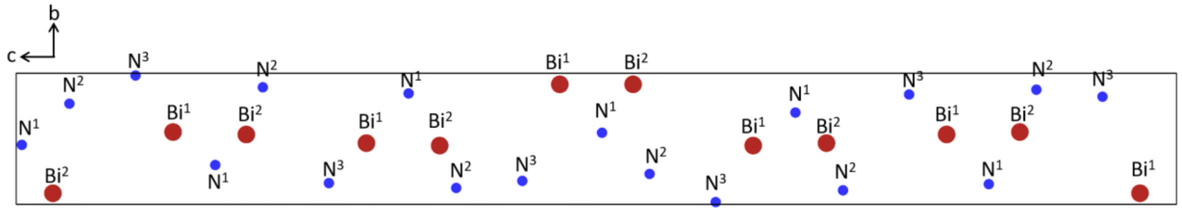

Figure S27. Distribution of Bi and N atoms of **$MHy_3Bi_2I_9$** in a unit cell.

Table S9. The atoms coordinate and coordinate the charge center in **$MHy_3Bi_2I_9$**.

Atoms	Atoms coordinate	Coordinate charge center
Bi^1	(0.5331,0.08471,0.03138) (0.08471,0.55161,0.86471) (0.55161,0.4669,0.69805) (0.44839,0.5331,0.19805) (0.91529,0.44839,0.36471) (0.4669,0.91529,0.53138)	(0.5,0.5,0.44805)
Bi^2	(0.46726,0.55165,0.13491) (0.91561,0.46726,0.30157) (0.55165,0.08439,0.96824) (0.08439,0.53274,0.80157) (0.53274,0.44835,0.63491) (0.44835,0.91561,0.46824)	(0.5,0.5,0.5515)
N^1	(0.546,0.7,0.32833) (0.7,0.154,0.16167) (0.154,0.454,0.995) (0.846,0.546,0.495) (0.3,0.846,0.66167) (0.454,0.3,0.82833)	(0.5,0.5,0.57833)
N^2	(0.875,0.768,0.954) (0.107,0.875,0.12067) (0.232,0.107,0.28733) (0.768,0.893,0.78733) (0.893,0.125,0.62067) (0.125,0.232,0.454)	(0.5,0.5,0.53733)
N^3	(0.017,0.838,0.23037) (0.838,0.821,0.0637) (0.821,0.983,0.89703) (0.162,0.179,0.5637) (0.983,0.162,0.73037) (0.179,0.017,0.39703)	(0.5,0.5,0.48037)

The polarization along the *c*-axis can be estimated to be:

$P_c = \lim \frac{1}{v} \sum q_i r_i$

$= (q_{Bi^1} r_{Bi^1} + q_{Bi^2} r_{Bi^2} + q_{N^1} r_{N^1} + q_{N^2} r_{N^2} + q_{N^3} r_{N^3})$

$= [(-1.5e \times 0.44805) \times 6 + (-1.5e \times 0.5515) \times 6 + (e \times 0.57833) \times 6 + (e \times 0.53733) \times 6 + (e \times 0.48037) \times 6] \times c / V$

$= [(0.58023) \times 1.6 \times 10^{-19} \times 67.038 \times 10^{-10} / (4435 \times 10^{-30})]$ (C/m^2)

$= 0.1403$ C/m^2

$= 14.03$ μC/cm^2

Table S10. The detection limit of the X-ray detection devices is based on $A_3Bi_2I_9$-type perovskites.

Structure	Space group	Electric field	Detection limit (nGy s^{-1})	Ref.
**$MHy_3Bi_2I_9$**	***$P6_1$***	**0**	**55**	**This work**
$Cs_3Bi_2I_9$	*P*6_3/*mmc*	50 V mm^{-1}	130	1
$MA_3Bi_2I_9$	*P*6_3/*mmc*	60 V mm^{-1}	83	2
$FA_3Bi_2I_9$	*P*6_3/*mmc*	180 V	200	3

Reference

[1] Y. Zhang, Y. Liu, Z. Xu, H. Ye, Z. Yang, J. You, M. Liu, Y. He, M. G. Kanatzidis, S. Liu, *Nat. Commun.* **2020**, *11*, 230

[2] Y. Liu, Z. Xu, Z. Yang, Y. Zhang, J. Cui, Y. He, H. Ye, K. Zhao, H. Sun, R. Lu, M. Liu, M. G. Kanatzidis, S. Liu, *Matter* **2020**, *3*, 180-196.

[3] W. Li, D. Xin, S. Tie, J. Ren, S. Dong, L. Lei, X. Zheng, Y. Zhao, W.-H. Zhang, *J. Phys. Chem. Lett.* **2021**, *12*, 1778.
